# Pelvic Organ Support in Animals with Partial Loss of Fibulin-5 in the Vaginal Wall

**DOI:** 10.1371/journal.pone.0152793

**Published:** 2016-04-28

**Authors:** Kathleen Chin, Cecilia Wieslander, Haolin Shi, Sunil Balgobin, T. Ignacio Montoya, Hiromi Yanagisawa, R. Ann Word

**Affiliations:** 1 Department of Obstetrics and Gynecology, Division of Female Pelvic Medicine and Reconstructive Surgery, University of Texas Southwestern Medical Center, Dallas, Texas, United States of America; 2 Department of Molecular Biology, University of Texas Southwestern Medical Center, Dallas, Texas, United States of America; Rutgers University -New Jersey Medical School, UNITED STATES

## Abstract

Compromise of elastic fiber integrity in connective tissues of the pelvic floor is most likely acquired through aging, childbirth-associated injury, and genetic susceptibility. Mouse models of pelvic organ prolapse demonstrate systemic deficiencies in proteins that affect elastogenesis. Prolapse, however, does not occur until several months after birth and is thereby acquired with age or after parturition. To determine the impact of compromised levels of fibulin-5 (Fbln5) during adulthood on pelvic organ support after parturition and elastase-induced injury, tissue-specific conditional knockout (cKO) mice were generated in which doxycycline (dox) treatment results in deletion of Fbln5 in cells that utilize the smooth muscle α actin promoter-driven reverse tetracycline transactivator and tetracycline responsive element-Cre recombinase (i.e., *Fbln5*^f/f^/SMA^++^-rtTA/Cre^+^, cKO). Fbln5 was decreased significantly in the vagina of cKO mice compared with dox-treated wild type or controls (*Fbln5*^f/f^/SMA^++^-rtTA/Cre^-/-^). In controls, perineal body length (PBL) and bulge increased significantly after delivery but declined to baseline values within 6–8 weeks. Although overt prolapse did not occur in cKO animals, these transient increases in PBL postpartum were amplified and, unlike controls, parturition-induced increases in PBL (and bulge) did not recover to baseline but remained significantly increased for 12 wks. This lack of recovery from parturition was associated with increased MMP-9 and nondetectable levels of Fbln5 in the postpartum vagina. This predisposition to prolapse was accentuated by injection of elastase into the vaginal wall in which overt prolapse occurred in cKO animals, but rarely in controls. Taken together, our model system in which Fbln5 is conditionally knock-downed in stromal cells of the pelvic floor results in animals that undergo normal elastogenesis during development but lose Fbln5 as adults. The results indicate that vaginal fibulin-5 during development is crucial for baseline pelvic organ support and is also important for protection and recovery from parturition- and elastase-induced prolapse.

## Introduction

Pelvic floor dysfunction is a prevalent disabling condition with suboptimal treatment. Women with pelvic organ prolapse (POP) suffer from urinary incontinence or retention, chronic pelvic pressure, fecal incontinence or obstruction, sexual dysfunction, social embarrassment and isolation. Up to 12.6% of women have surgery for POP in their lifetime [1]. Regrettably, of 400,000 operations performed for incontinence and prolapse per year, 116,000 are repeat operations (i.e., 29%) [2, 3].

Little is known regarding the pathogenesis of POP. Mouse models indicate that compromise of elastogenesis [4–6], together with upregulation of proteases [7, 8] play a role in the pathogenesis of POP. Specifically, *LOXL1* knockout mice develop POP both as a function of age and after parturition [5, 9]. Mice deficient in fibulin-5 (Fbln5) develop prolapse as a function of age even without vaginal delivery (90% by 6 months of age) [4]. *Fbln3* knockout mice also develop prolapse as a function of age (*27%* [10]). Interestingly, although these animal models exhibit elastinopathies at birth, POP does not develop until later in life [11]. This observation suggests that abnormal elastic fibers, alone, may not be sufficient to induce prolapse, but that other factors acquired during parturition and aging, together with abnormal elastic fibers, lead to prolapse. One of these factors is likely activation of MMP-9. MMP-9 activity is increased dramatically (i) after vaginal delivery [8], (ii) after vaginal distention injury [7], and (iii) several weeks prior to the onset of prolapse in *Fbln5*^*-/-*^ mice [11] and (iv) after estrogen withdrawal [12]. Moreover, MMP-9 is strikingly upregulated in the vaginal wall of women with prolapse [13–15] and has recently shown to be genetically linked [16]. Elastic fiber defects alone, however, are insufficient to activate MMP-9 because increased activity was not observed in aorta or skin from *Fbln5*^*-/-*^ animals [14].

The two major risk factors for POP in humans are history of vaginal delivery and aging [3, 17]. Fbln5 has been shown to be downregulated in pelvic floor connective tissues of women with prolapse compared with controls [18]. Further, Fbln5 is cleaved with age (29), and protease inhibitors that limit elastic fiber degradation are lost in the vaginal wall of humans and mice with age [18]. Thus, loss of Fbln5 with age may lead to increased MMP-9 in connective tissues of the pelvic floor. In most organs, elastogenesis is complete after development with little or no elastic fiber renewal during adulthood. The female reproductive tract is unique with evidence of continuous remodeling and regeneration of elastic fibers [19, 20]. Hence, downregulation of vaginal Fbln5 may also lead to compromised renewal of elastic fibers in the vaginal wall. Here, we hypothesized that acquired loss of Fbln5 after the developmental time period (e.g., during pregnancy and after parturition or with aging) may result in POP due to compromise of elastogenesis or failure to suppress MMP-9 in connective tissues of the pelvic floor.

## Materials and Methods

### Generation of conditional tissue-specific Fbln5 deficient mice

**Fig 1:**
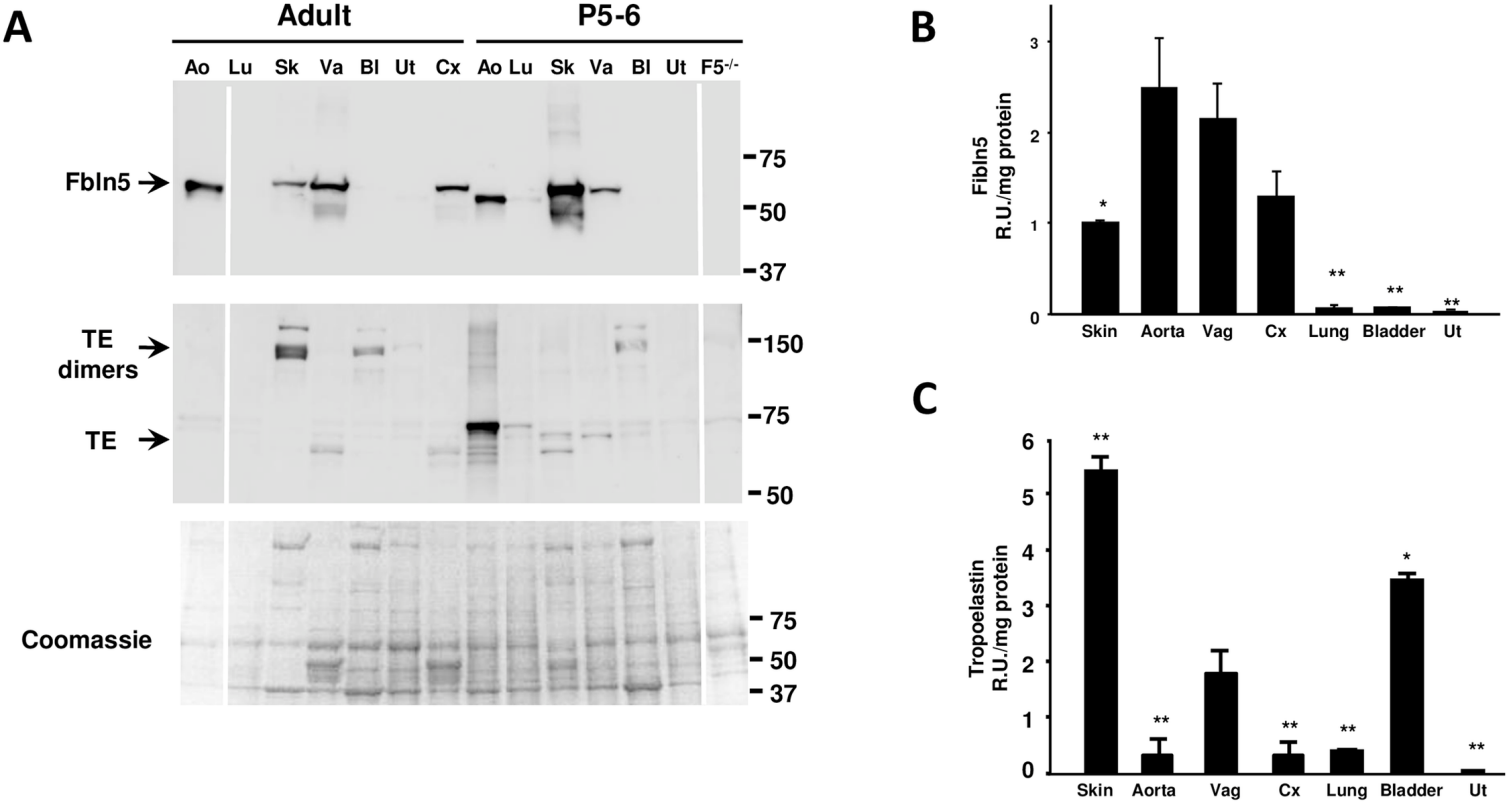
**A. Targeting strategy.** The exons 1a through 4 are numbered. Wild-type, targeting vector, targeted alleles (*neo-loxP*, *loxP*, *and KO*) are shown. *FLPe*-mediated excision removes a *Neo*^*r*^ cassette to generate *Fbln5*^*loxP*^ allele and Cre-mediated excision removes exons 1a and 1b to generate a null allele. DTA: Diphtheria Toxin A; H: Hind III; Xb: Xba I; Xo: Xho; Kp: Kpn I; B: Bam HI; RI: Eco RI, Sc: Sac I. **B.** Confirmation of mutant alleles by genomic PCR. Amplification with primer set A distinguishes *Fbln5*^*loxP*^ allele from wild-type (+/+) allele. Primer set B only amplifies *Fbln5*^*KO*^ allele. * non-specific band.

To control the temporal expression of Cre recombinase in vivo, we employed an inducible tetracycline (Tet) Cre-*lox*P system. Transgenic mice harboring rtTA under the control of the smooth muscle α actin promoter (obtained from Dr. Mike Shipley, Washington University, St. Louis, MO) (hereafter referred to SMA^++^) were mated with tetO-Cre transgenic mice (obtained from Dr. Andreas Nagy, Mount Sinai Hospital, Toronto) to generate double transgenic mice. In these mice, administration of doxycycline results in expression of Cre recombinase in vaginal stromal cells, myofibroblasts, and smooth muscle cells. The targeting approach is illustrated in Fig 1. Two *lox*P sites flanked alternative exons 1a and 1b of Fbln5 and a neomycin resistant gene (*Neo*^*r*^), which is flanked by FRT sites. Lengths of the short, middle and long arms are approximately 1.5 kb, 1 kb and 7.0 kb, respectively. A targeting vector was constructed using 129SvEv genomic DNA and the linearized vector was electroporated into SM-1 ES cells as previously reported [21]. A mutant allele containing the *Neo*^*r*^ cassette and *lox*P sites (*Fbln-5*^neo-loxP^) was obtained by homologous recombination using positive selection with neomycin (G418) and negative selection with diphtheria toxin A (DTA). ES cells containing the correct targeted allele were then injected into blastocysts obtained from C57/Bl6 females. The resulting chimeric males were bred to C57Bl/6 females for germ line transmission.

*Fbln5*^*-/-*^ and *Mmp*^*-/-*^ mice were previously described [14, 22] and kept on a 12 h/12 h light/dark cycle. All animal experimental procedures were reviewed and approved by the Institutional Animal Care and Use Committees of the University of Texas Southwestern Medical Center and sacrificed by an overdose of Avertin (tribromoethanol, 500 mg/Kg) followed by exsanguination.

### Tissue acquisition and processing

Lung, skin, aorta, bladder, uterus and vagina from adult rats, embryonic females (E20-22) and neonatal rats (P1-2 and P5-6) were dissected, flash frozen in liquid N_2_ and stored at -80 C until processing. Vaginal tissues were obtained from mice by dissecting the entire vaginal tube from the cervix to the perineum. Rings of vaginal tissue were obtained either from the mid-vagina or from the elastase-injected posterior vagina and mounted in the transverse orientation for histology. The tube was then opened longitudinally, epithelium removed by scalpel, and 1/3 of the remaining vaginal muscularis was frozen in liquid N_2_ for zymography and 2/3 for immunoblotting.

### Homogenization

Due to the small amounts of tissue from embryonic and postnatal rats, tissues were homogenized in 16 mM potassium phosphate pH 7.8, 0.12 M NaCl and 1 mM EDTA and 6 M urea for developmental studies. Thereafter, the homogenates were extracted overnight to obtain extracellular matrix proteins for immunoblot analysis. In the case of the mouse vaginal wall, frozen vaginal tissue was pulverized with a liquid nitrogen-chilled mortar and pestle. Tissue powder was then homogenized in basic buffer containing protease inhibitors (16 mM potassium phosphate pH 7.8, 0.12 M NaCl, 1 mM EDTA, 0.1 mM PMSF, 10 μg/ml pepstatin A, and 10 μg/ml leupeptin), and centrifuged at 10,000x *g*. The supernatant was then removed and the previous homogenization step repeated after resuspending the remaining tissue pellet in basic buffer. After removal of the second supernatant, the remaining tissue pellet was suspended in urea buffer (6.0 M urea in above basic buffer), homogenized, and placed on a rotating rack for overnight extraction at 4°C. Thereafter, the samples were centrifuged (10,000x *g* x 30 min) and the supernatant removed. Protein concentrations were determined using a BCA protein assay and standard curves of BSA in appropriate buffers.

### Immunoblot analysis

Total protein (20 μg/lane) was applied to 4–20% Criterion gradient polyacrylamide gels (BioRad, Hercules, CA), separated by electrophoresis, and transferred to nitrocellulose membranes overnight at 4 C. To ensure equal protein loading, identical gels were run side-by-side for Coomassie staining. Nitrocellulose membranes were placed in blocking buffer (10 mM Tris HCl, pH 7.5, 0.15 M NaCl, 0.1% Tween 20, 2% nonfat powdered milk, 0.01% thimerosol) for 1 hour at 37 C and incubated with primary antibody for 1 hour at 30 C. Membranes were then washed with TBST (10 mM Tris HCl, pH 7.5, 0.15 M NaCl, 0.1% Tween 20) for 5 min x 3, an enhanced detergent wash (TBST, NP 40 0.05%, 3 mM sodium deoxycholate, and sodium dodecyl sulfate 0.1%) for 7 min x 3, and again with TBST for 5 min x 3. Thereafter, the blot was incubated with second antibody (goat IgG-HRP conjugate 1:8000) at room temperature for 1 hour. The membrane wash protocol was repeated, followed by incubation with Western Lightning Chemiluminescence Reagent Plus (Perkin-Elmer, Boston, MA). Signal strength was captured using a Fujifilm FLA 5100 image capture system. Protein band density was calculated using Image J software (ImageJ 1.46r, NIH) and normalized to total protein loaded quantified on Coomassie-stained gels. The relative signal strength per μg of urea-extracted protein was calculated and normalized to an extract which served as an standard on each blot. Rabbit anti-rat FBLN5 (BSYN1923) was used at 1:250 dilution. Rabbit anti-mouse tropoelastin was obtained from Elastin Products (Owensville, MO).

### Histomorphology

Vaginal rings were fixed in neutral buffer formalin (10%). Tissues were subsequently processed and embedded in paraffin blocks. Cross-sections of each vaginal ring were stained with Hematoxylin and Eosin or Hart’s stain. Images of each section were captured and analyzed using a Nikon E1600 microsocpe and Nikon NIS Elements AR software (Melville, NY). Adjacent images (6–10 images per specimen) of Hart’s stained sections at 400X were converted to grey scale and calibrated. The binary set function was used and threshold set to 100 for all sections to outline all elastic fibers. The region of interest (ROI) was outlined to exclude epithelium. Fractional area of elastic fibers relative to ROI, length, circularity and elongation and number of fibers in each section were computed.

### Zymography

Gelatin zymography was performed as described [10]. Briefly, tissues were homogenized with buffer (10mM Tris, 150mM NaCl, 10mM CaCl2, and 0.1% Triton X-100, pH7.4), centrifuged at 16,000 g for 15 min at 4°C, and the supernatant was used for assay. Protein (5 μg) was applied to 10% gelatin polyacrylamide minigels in standard sodium dodecyl sulfate loading buffer containing 0.1% sodium dodecyl sulfate. After electrophoresis, gels were soaked in renaturing buffer (2.7% Triton X-100 in distilled water) in a shaker for 30 min with one change after 30 min to remove sodium dodecyl sulfate. Gels were soaked in assay buffer (50 mM Tris, 200 mM NaCl, 10 mM CaCl_2_, 0.05% Brij 35, pH 7.5) for 16 h at 34°C and stained with Coomassie Brilliant Blue-R 250 in 50% methanol and 10% acetic acid followed by destaining with 25% methanol and 7% acetic acid. Areas of lysis were analyzed using a Fuji LAS 3000 image analysis system (Fujifilm Life Science, Tokyo, Japan).

### Statistical Analysis and Power Calculation

Statistical comparisons between groups were conducted by Student’s t test or, for multiple groups, one-way ANOVA using Dunnet’s comparison of means. P values ≤ 0.05 were considered significant. Based on the variability determined from pilot studies, we used a 2:1 disease: control ratio to estimate a sample size of 5 controls and 9 prolapse to detect a 2-fold increase in elastase-induced prolapse development with a power of 0.80 and an alpha of 0.05.

## Results

### Expression of tropoelastin and Fbln5 during development and adulthood

**Fig 2:**
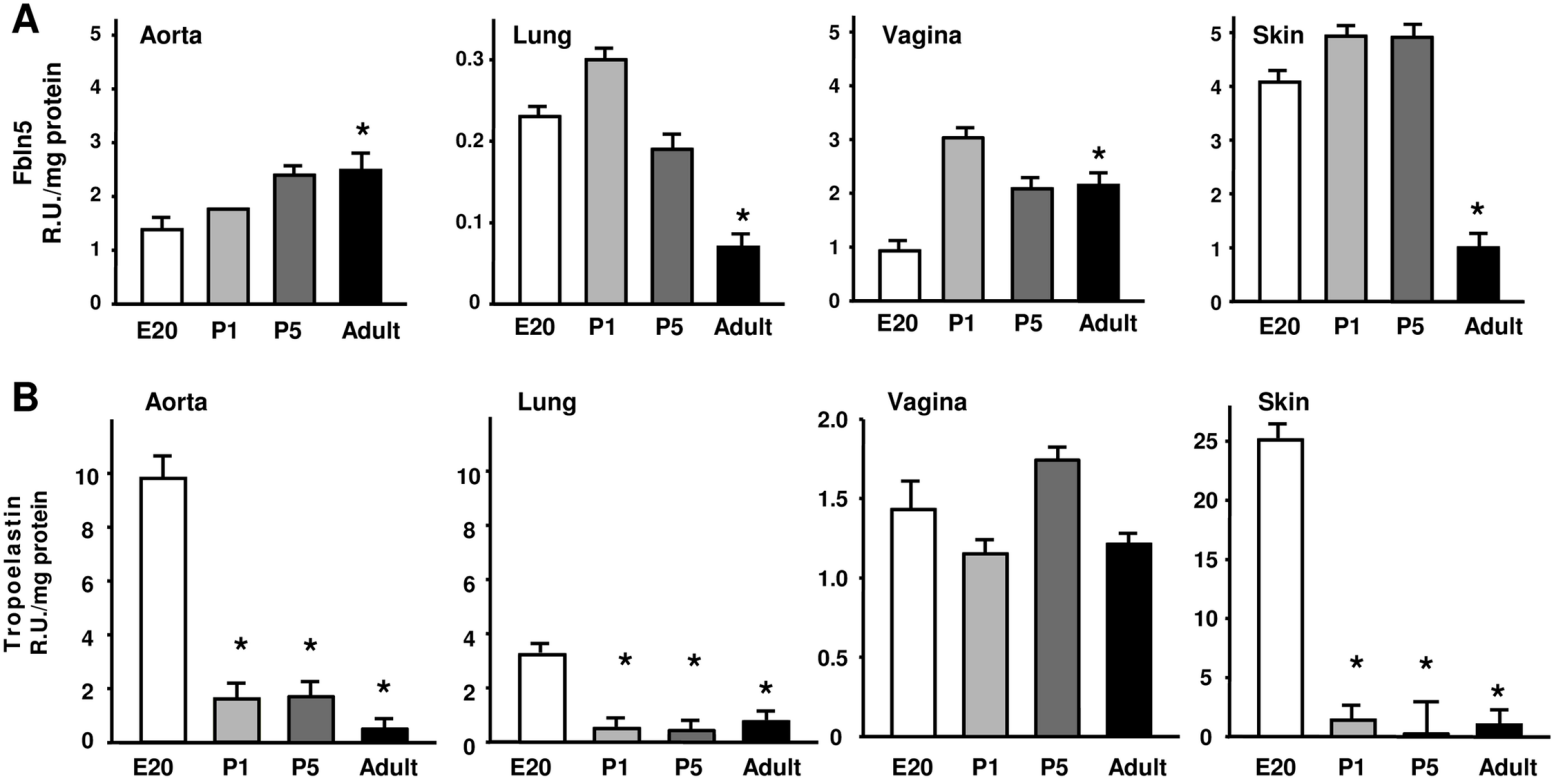
Expression of Fbln5 and urea-extractable tropoelastin (TE) in tissues from adult and postnatal (P5-6) rats. **A. Representative immunoblot of Fbln5 (upper blot) and tropoelastin (TE, lower) of adult and postnatal tissues**. Coomassie-stained gel of protein extracts shown as an index of protein loading. Tissues were homogenized directly in urea buffer and extracted overnight. **Ao**, aorta; **Lu**, lung; **Sk**, skin; **Va**, vagina; **Bl**, bladder; **Ut**, uterus; **Cx**, cervix. Quantification of Fbln5 (**B**) and tropoelastin (**C**) in urea extracts of adult tissues. Data represent mean ± SEM of 3–4 tissues in each group. *p<0.05; **p<0.05 compared with vagina as the control, ANOVA with Dunnett’s post hoc testing

Elastin is produced in early development, reaching peak levels in the third trimester of fetal life [23, 24]. In most tissues, elastic fiber synthesis steadily decreases during early postnatal development and ceases in adults. Recent evidence, however, suggests that elastic fiber renewal occurs in the adult female reproductive tract especially after parturition [20, 25]. Thus, our initial experiments were conducted to compare levels of Fbln5 and tropoelastin in the vagina with that of other adult elastic tissues, and to compare expression patterns of these two proteins in various tissues from early postnatal development until adulthood. Rats were used as an experimental model. Matrix-bound Fbln5 is solubilized by extraction in 6 M urea. Although immature newly-synthesized tropoelastin is solubilized in urea, fully mature cross-linked elastic fibers are not. In resting adult connective tissues, Fbln5 was enriched in aorta, skin, vagina and cervix but low to nondetectable in lung, bladder, and uterus (Fig 2A and 2B). On P5-6, Fbln5 expression was similar to that in adults. Interestingly, however, the molecular size was decreased in the postnatal time period, suggesting possible changes in glycosylation or alternative splicing of the amino terminus in the adult aorta. In skin, Fbln5 expression was decreased in the adult relative to P5-6, but Fbln5 expression was maintained in the adult vagina. Soluble extracted tropoelastin expression was evaluated in urea extracts from adult and postnatal tissues (Fig 2A and 2C). Extracted tropoelastin monomers were poorly expressed in the adult aorta relative to tropoelastin dimers extracted from skin and bladder. Interestingly, urea-extracted tropoelastin was highly expressed in the aorta and lung on P5-6, but not in the adult aorta or lung, suggesting that elastin turnover (or newly synthesized tropoelastin) is complete in adult aortic and lung tissue. In contrast, solubilized tropoelastin was expressed in both adult and postnatal vaginal tissues, suggesting the possibility of continuous formation of elastic fibers.

**Fig 3:**
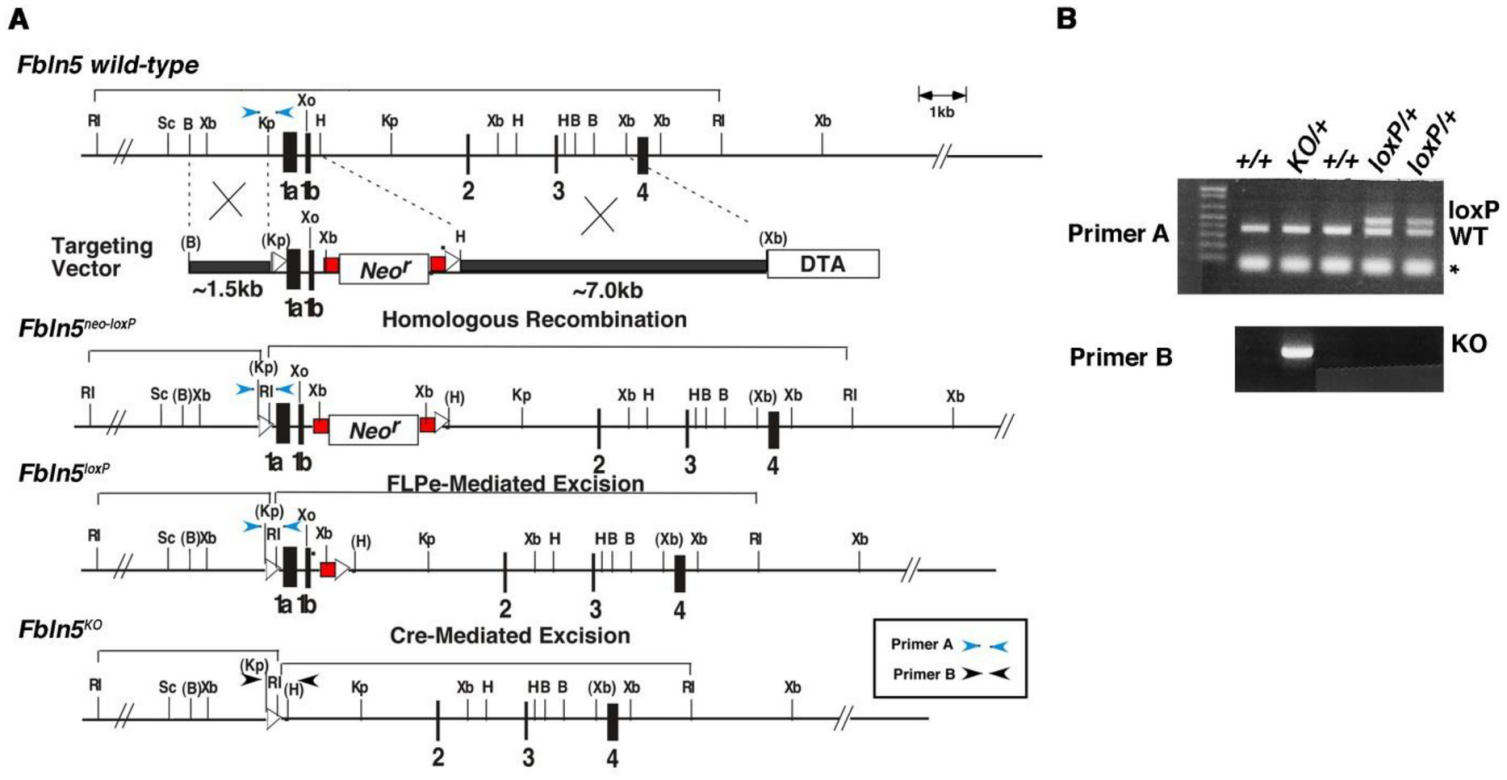
Expression of Fbln5 and urea-extractable soluble tropoelastin (TE) in tissues as a function of development in rats. Quantitative amounts of Fbln5 (PANEL A) and extracted tropoelastin **(B)** normalized to coomassie-stained protein in aorta, lung, vagina, and skin. Data represent mean ± SEM of 3–4 tissues in each group. **RU**, Relative Units, **E20**, embryonic day 20, **P1**, postnatal day 1; **P5**, postnatal day 5 *p<0.05 ANOVA with Dunnett’s post hoc testing with E20 as the control

A more detailed time course of Fbln5 and tropoelastin protein content was determined during development (Fig 3). Fbln5 content in the aorta increased from E20 to P5 and was maintained in the adult (Fig 3A). In the lung, Fbln5 content was less than in the aorta with maximal levels on P1 decreasing to almost undetectable levels in adults. Vaginal Fbln5 content was similar to that of the aorta and was maintained at high levels in the adult. Although embryonic and postnatal skin was also enriched in Fbln5, Fbln5 decreased significantly in adult skin (Fig 3A). Fbln5 was poorly expressed in postnatal (E20-P5) and adult bladder and uterus (Fig 2A and data not shown). Although Fbln5 increased during development with maximal levels in the adult aorta, the opposite trend was observed for tropoelastin (Fig 3B). In the early postnatal aorta, extractable tropoelastin was highly expressed falling to almost nondetectable levels in the adult. Likewise, in lung and skin, extractable tropoelastin content decreased after birth. This pattern of extractable tropoelastin protein in adult connective tissues differed significantly in the vagina in which tropoelastin remained extractable throughout embryonic and adult life. Although Fbln5 was poorly expressed in the bladder, TE dimers were extracted in both postnatal and adult bladder (Fig 2A and 2C).

Taken together, these studies indicate that in most connective tissues, extractable tropoelastin decreases after development. Likewise, in the lung and skin, Fbln5 expression decreases after development. In contrast, Fbln5 is highly expressed in the vagina during development and in the adult. Further, tropoelastin remains extractable during all stages of development. It has been shown that elastogenesis plays a crucial role in maintenance of pelvic organ support in female mice [4, 5, 10]. Our data demonstrating maintenance of both Fbln5 and extractable tropoelastin proteins in the matrix of the adult vaginal wall support the hypothesis that elastic fiber turnover may be unique in the vagina compared with other connective tissues. This adaptation may facilitate renewal of elastic fiber networks and thereby contribute to recovery of vaginal connective tissues after childbirth or surgical injury.

### Generation of tissue-specific Fbln5 knockout mice

To test the hypothesis that synthesis of Fbln5 in the vagina is crucial for maintenance of pelvic floor support after completion of elastogenesis, tissue-specific conditional knockout (cKO) mice were generated in which doxycycline treatment would result in deletion of Fbln5 in cells that utilize the smooth muscle α actin promoter (i.e., vaginal stromal cells, myofibroblasts, and smooth muscle cells, Fig 1).

**Fig 4:**
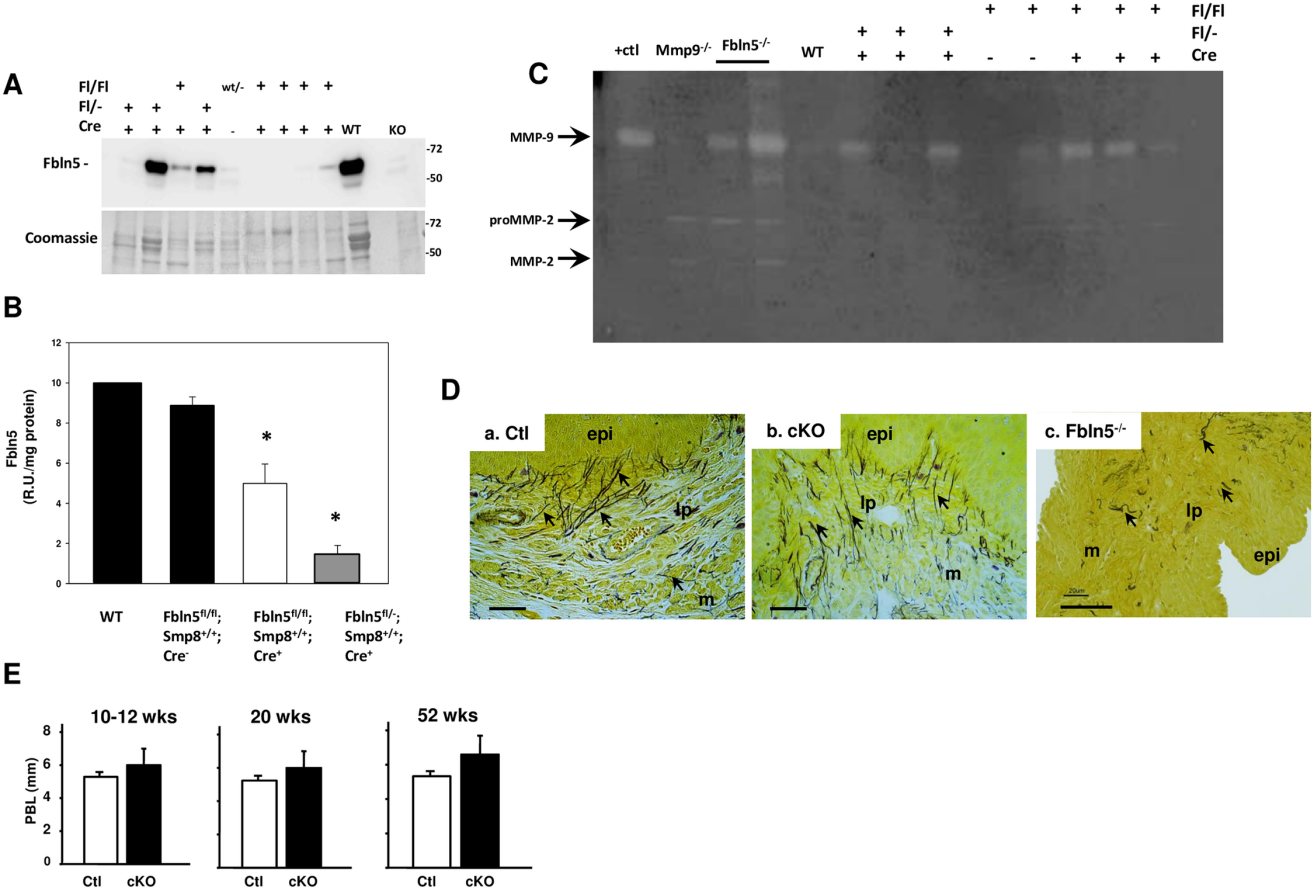
Expression of Fbln5 in targeted mice. rtTA/*Fbln5*^f/f^/SMA^++^/Cre^-^, rtTA/*Fbln5*^f/-^/SMA/Cre+, or rtTA/*Fbln5*^f/f^/SMA^++^/Cre^+^ mice were treated with doxycycline as outlined in Materials and Methods. Thereafter, Fbln5 was quantified in the fibromuscular layer of the vaginal wall. **A.** Representative immunoblot and coomassie stained protein gel. **B.** Quantification of Fbln5 in vaginal urea extracts from *Fbln5*^f/f^/SMA^++^/Cre^-^ (n = 7, solid bar), *Fbln5*^f/f^/SMA/Cre+ (n = 9, open bar), or *Fbln5*^f/-^/SMA^++^/Cre+ (n = 7, grey bar) normalized to protein content. Tissue extracts from wild type (**WT)** animals were used as a standard on each gel. *p < 0.05 compared with Cre- animals, ANOVA followed by Dunnett’s test using WT as control. **C.** Gelatin zymography of soluble protein extracts from *Fbln5*^f/f^/SMA^++^/Cre^-^, *Fbln5*^f/-^/SMA/Cre+, or *Fbln5*^f/f^/SMA^++^/Cre+. Purified proMMP (**+ctl**), protein from **Fbln5**^**-/**-^, and **Mmp9**^**-/-**^ vaginal tissues were used as positive and negative controls. **D.** Hart’s stain of mid-vagina from *Fbln5*^f/f^/SMA^++^/Cre^-^ (**a. Cre-**) or *Fbln5*^f/f^/SMA^++^/Cre+ (**b. Cre+**) mice in basal conditions. Vaginal wall from age-matched Fbln5^-/-^ mouse shown in **c** (**Fbln5**^**-/-**^). Arrows indicate elastic fibers. **epi**, vaginal epithelium; **lp**, lamina propria; **m**, muscularis. Bar = 40 μm. Note decreased magnification in **c** to illustrate paucity of fibers. **E.** Effect of Fbln5 cKO on spontaneous development of prolapse as a function of age. Perineal body length was measured at 10–12, 20, and 52 weeks of age in *Fbln5*^f/f^/SMA^++^/Cre^-^ (**Ctl**, n = 3) and *Fbln5*^f/f^/SMA^++^/Cre^+^ (**cKO**, n = 4) mice. Magnitude of bulge did not differ among genotypes (not shown). All animals were treated with doxycycline at 6 weeks of age.

In these animals, doxycyline treatment (2 mg/ml in 5% sucrose water x 3 weeks) resulted in significant loss of Fbln5 protein levels in the vagina relative to that of vaginal tissues from doxycycline-treated wild type or *Fbln5*^f/f^/SMA^++^/Cre^-/-^ mice (negative controls) (Fig 4A and 4B). Loss of Fbln5 was variable among animals. Whereas some doxycycline-treated cKO (*Fbln5*^f/f or f/-^/SMA^++^/Cre^+/+^) mice (~10%) exhibited vaginal Fbln5 levels similar to that of wild type (for example, compare lanes 2, 3, and WT), Fbln5 was barely detectable in 33% and moderately suppressed in the rest. Overall, Fbln5 levels were 50% and 20% that of controls in Fbln5^fl/fl^ and Fblin5^fl/-^ animals, respectively (Fig 4B). cKO animals were healthy and exhibited normal breeding and fecundity. Like *Fbln5* heterozygote mice [7], partial loss of Fbln5 was accompanied by upregulation of vaginal MMP-9 (Fig 4C). Conditional knockdown of Fbln5 did not alter elastic fiber morphology (Fig 4D, Table 1). Virginal cKO and negative controls were observed for one year with weekly measurements of pelvic organ support. As shown in Fig 4E, pelvic organ support (perineal body length shown) did not differ between nonpregnant controls and cKO (*Fbln5*^f/f^/SMA^++^-rtTA/Cre^+^) animals. Specifically, spontaneous prolapse was not detected up to one year after doxycycline treatment (Fig 4E).

**Table 1:** Quantification of elastic fibers and morphology in vaginal tissues from Cre- and Cre+cKO animals.

Group	Elastic fiber area (%)	Elastic fiber length (μm)	Fibers > 5 μm	Maximal fiber length (μm)
Baseline Cre^-^n = 3	8.9 ± 0.59	5.36 ± 0.7	10 [8,12]	28.8 ± 10.8
Baseline Cre^+^n = 3	9.2 ± 0.51	5.42 ± 0.20	8 [6,9]	29.0 ± 3.4
Cre^-^ Elastasen = 3	9.6 ± 0.21	4.99 ± 0.14	12 [9,18]	18.8 ± 4.2
Cre^+^ Elastasen = 4	4.9 ± 0.76*	2.31 ± 0.51*	4 [1,2]*	13.6 ± 3.6*

Data represent mean ± SEM, except number of fibers which is expressed as median [range].

*p < 0.05 compared with Cre- elastase-injected animals.

Together, these data indicate that partial loss of Fbln5 in the vaginal wall resulted in modest upregulation of vaginal MMP-9, normal elastic fibers in the vagina, and no prolapse, suggesting that, under normal physiological conditions, knockdown of Fbln5 after elastogenesis during development results in a normal phenotype but increased MMP-9 in the vagina.

### Effect of parturition on pelvic organ support in cKO mice

**Fig 5:**
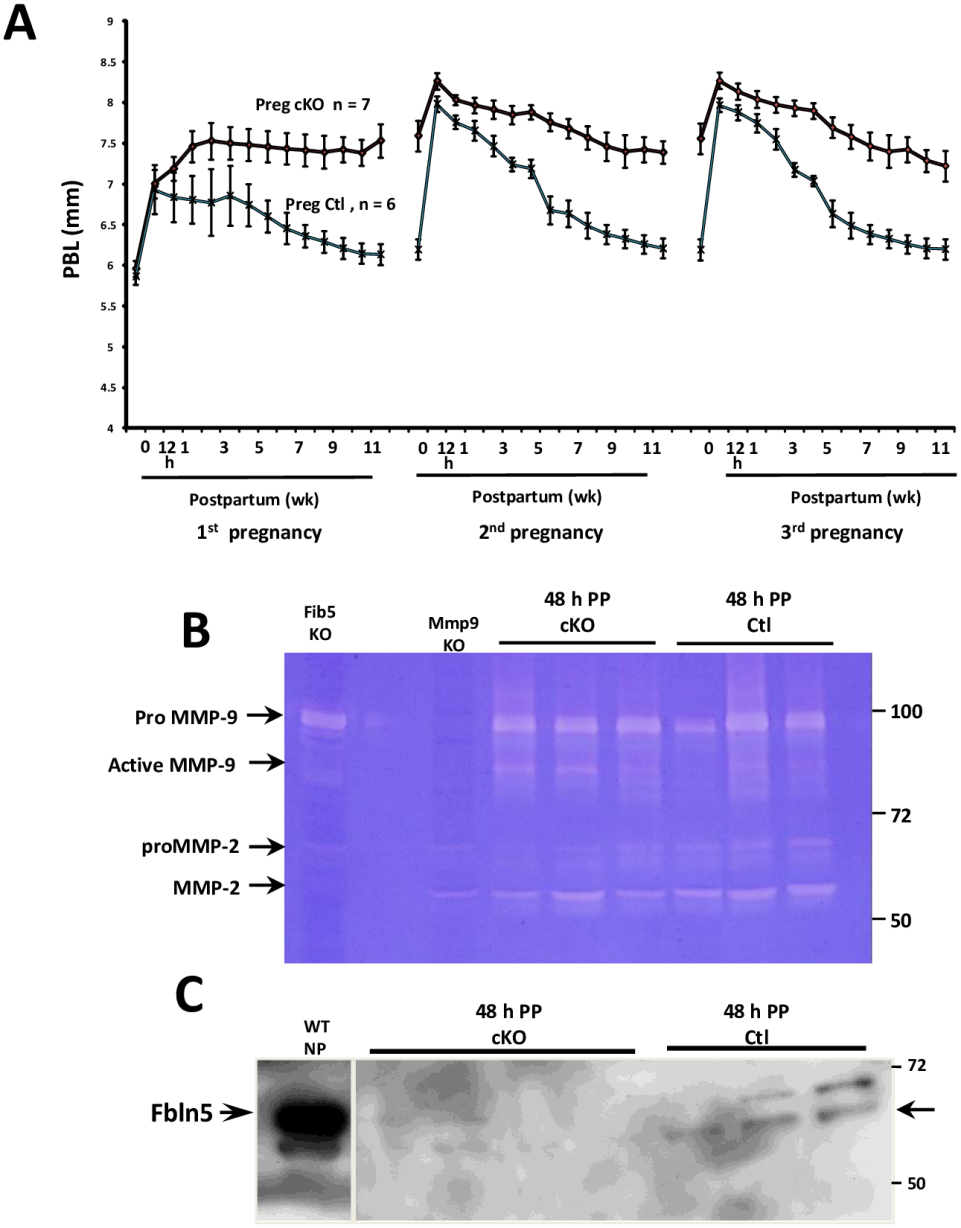
Effect of pregnancy and vaginal delivery on *Fbln5* cKO mice. **A.** Perineal body length was measured at time 0 (prior to pregnancy) and 12 h to 12 wks postpartum in *Fbln5*^f/f^/SMA^++^/Cre^+^ (**Preg cKO**, n = 7) and *Fbln5*^f/f^/SMA^++^/Cre^-^ mice (**Preg Ctl**, n = 6) during 3 pregnancies. **B.** Gelatin zymography of vaginal tissue extracts from *Fbln5*^f/f^/SMA^++^/Cre^+^ (**cKO**) and *Fbln5*^f/f^/SMA^++^/Cre^-^ (**Ctl**) mice 48 h postpartum. Vaginal tissue extracts from *Fblin5*^*-/-*^ (**Fib5 KO**) and *Mmp9*^*-/-*^ (**MMP9 KO**) mice were used as positive and negative controls. Lane 2 is blank. **C.** Immunoblot of Fbln5 in urea extracts of vaginal muscularis from wild type nonpregnant control **(WT NP**), **cKO**, and **Ctl** animals 48 h postpartum. Arrow denotes cleaved form. All animals were treated with doxycycline at 6 weeks of age, mated, and tissues collected after the first pregnancy.

Next, we tested the hypothesis that normal Fbln5 levels are crucial for inhibition of MMP-9 and re-establishment of elastic fiber networks in the vaginal wall after parturition. cKO and negative controls were treated with doxycycline at 6 weeks of age. After 3 weeks, animals were mated and MOPQ measurements [11] were conducted before and after the first, second, and third pregnancy by examiners blinded to genotype (Fig 5A). In negative controls, perineal body length (PBL) increased significantly after delivery but declined to supra-baseline values within 6–8 weeks. These transient increases in perineal body length postpartum were more dramatic in subsequent pregnancies, even in negative controls. Interestingly, however, in cKOs, PBL measurements also increased significantly in the early postpartum period, but, unlike controls, parturition-induced increases in PBL did not recover to baseline. Rather, PBL remained increased in cKOs for 12 weeks postpartum. Although overt prolapse did not occur in cKO animals, even after three pregnancies, these results suggest that Fbln5 is important for full recovery of pelvic support from vaginal delivery.

To gain insight regarding the potential molecular mechanisms of this poor recovery from parturition in cKO animals, cKO and Cre- control vaginal tissues were collected 48 h after the first delivery and analyzed for MMP9 activity and Fbln5 levels (Fig 5B). Although pro-MMP-9 and MMP-2 levels were comparable between cKO and controls, active MMP-9 was increased in postpartum cKO animals (Fig 5B). Previously, we found that Fbln5 protein levels are suppressed in the vaginal wall of pregnant animals with partial recovery 48–72 h postpartum [4]. To determine if recovery was similar in cKO animals, Fbln5 levels were quantified in urea extracts of vaginal muscularis from postpartum cKO and negative controls using immunoblotting (Fig 5C). As expected, vaginal Fbln5 protein levels were decreased postpartum compared with nonpregnant controls. In contrast with resting nonpregnant animals, Fbln5 appeared as two immunoreactive proteins in postpartum animals consistent with full-length and a cleaved product of Fbln5 that does not support elastic fiber assembly [26]. Interestingly, during this vulnerable time during which the vagina recovers from parturition, protein levels of Fbln5 were virtually nondetectable in cKO animals even with overexposed immunoblots. Since Fbln5 was previously shown to inhibited fibronectin-induced upregulation of MMP-9 in vaginal stromal cells [14], this severe compromise of postpartum vaginal Fbln5 may contribute to upregulation of MMP-9 and compromised recovery of vaginal support after parturition.

### Effect of elastase-induced injury of the vaginal wall

**Fig 6:**
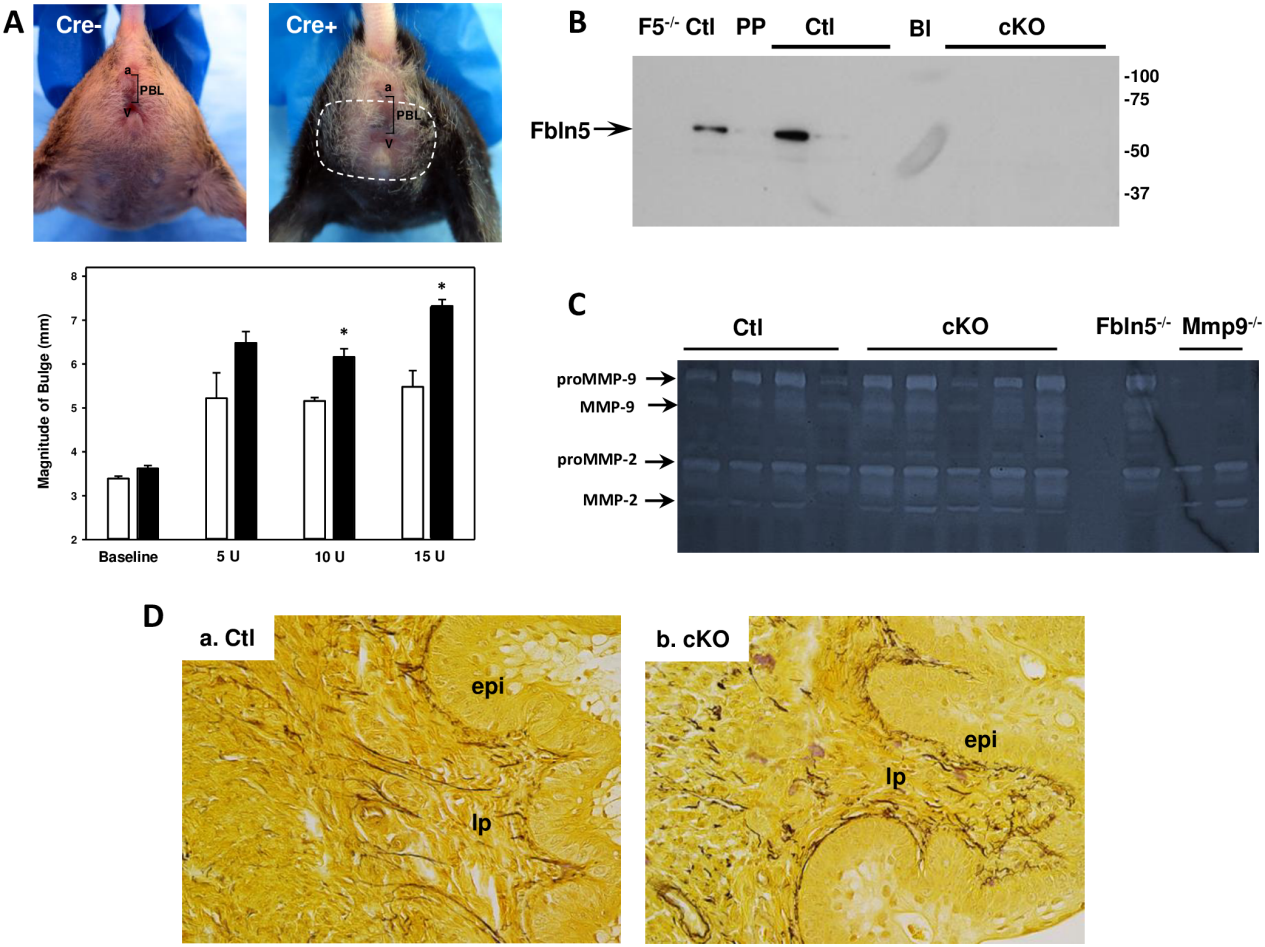
Effect of elastase injection on *Fbln5* Cre+cKO mice. **A.** Purified porcine elastase was injected into the posterior vaginal wall in *Fbln5*^f/f^/SMA^++^/Cre^-^ (**Cre-**, **open bar**, n = 5) and *Fbln5*^f/f^/SMA^++^/Cre^+^ or *Fbln5*^f/-^/SMA^++^/Cre^+^ (**Cre**^**+**^, **solid bar**, n = 9). MOPQ measurements were recorded 48 h after 5, 10, and 15 U of elastase. Since effects of elastase did not differ among *Fbln5*^f/f^/SMA^++^/Cre^+^ (n = 4) compared with *Fbln5*^f/-^/SMA^++^/Cre^+^ (n = 5), data were combined as mean ± SEM. Magnitude of bulge shown. *p < 0.05 compared with Cre-. **B**. Immunoblotting of Fbln5 in urea extracts from elastase-injected vaginal muscularis of Cre- and Cre+cKO mice. F5^-/-^, negative control; Ctl, nonpregnant wild type; PP, 48 h postpartum wild type control indicating decrease in Fbln5. Coomassie gel indicated even loading (not shown). **C.** Gelatin zymography of Ctl and cKO vaginal extracts from elastase-treated mice. **D.** Hart’s stain of posterior vaginal wall of elastase-injected Ctl (**a**) or cKO (**b**) mice.

Since mice do not experience traumatic injury during parturition, we sought to determine the impact of decreased levels of Fbln5 in the vaginal wall on recovery of pelvic organ support after elastase injury. This approach is accepted as a model system for connective tissue injury for aortic aneurysms [27–34] and emphysema [35–40]. Specifically, porcine pancreatic elastase (5 U in 100 μl) was injected transvaginally in 5 Cre^**-**^ and 9 cKO animals after DOX treatment. MOPQ measurements were recorded at 48 h. If prolapse was not obvious within 48 h by MOPQ examination, elastase was increased by 5 U. After MOPQ measurements, tissues were then collected 48 h from the distal posterior vagina after injection of 15 U transvaginally for assessment of Fbln5 content, MMP9 activity, and elastic fiber morphology (Fig 6). After the first injection, the magnitude of bulge increased in both Cre- controls (Ctl) and cKO animals but did not differ between the two groups. After the second injection (10 U), however, the magnitude of bulge was significantly increased in cKO which continued to increase after 15 U (Fig 6A and 6B). Perineal body length measurements also increased approaching statistical significance (6.5 ± 0.4, Ctl, compared with 7.2 ± 0.4 mm, cKO, *p* = 0.09). If bulge was >95% CI of control animals, the animal was diagnosed with prolapse. In elastase-treated cKO animals, 8 of 9 developed prolapse (Stage 3, n = 3; Stage 2, n = 5). Although not altered in one Ctl mouse, Fbln5 content was decreased but detectable in vaginal tissues from Ctl animals injected with elastase (Fig 6B). In contrast, Fbln5 was not detectable in injected cKO animals. MMP-9 activity was increased in all elastase-injected vaginal tissues with no appreciable differences between Ctl and cKO (Fig 6C).

The impact of elastase on elastic fiber morphology was distinct between Ctl and cKO (Fig 6D). In Ctl animals, elastic fiber length remained similar to that of untreated animals (Fig 6D, Table 1). However, branches of the fibers reaching the basement membrane of the epithelium were absent with short remnants of fibers lining the subepithelium (Fig 6D). Although area of elastic fibers, circularity, and elongation were similar to noninjected controls, as expected, maximal fiber length was decreased modestly (Table 1). Treatment of cKO animals resulted in significant loss of elongated elastic fibers (Fig 6D). Like Ctl animals, a layer of transected fibers lined the subepithelium. Elastic fiber area, length, and elongation were decreased significantly in elastase-injected cKO animals. Further, the number of fibers > 5 μm and maximal fiber length were decreased (Table 1). Results indicate that cKO, but not Ctl, develop significant prolapse, loss of Fbln5, and reduced elastic fiber integrity after elastase injection suggesting that vaginal Fbln5 is crucial for protection from protease-induced degradation of elastic fibers.

## Discussion

To understand the function of Fbln5 in pelvic organ support after parturition or injury in adults that have baseline normal elastic fibers, we generated mice deficient in Fbln5 in alpha-SMA-positive vaginal stromal cells and smooth muscle cells. We expected that failed up-regulation of Fbln5 in the vagina after injury or parturition would lead to the development of vaginal/uterine prolapse due to a failure of proper elastic fiber remodeling (i.e., rebuilding of the elastic fiber network). Our present study, however, showed that compromise, but not complete loss, of Fbln5 in the vaginal wall led to (i) subclinical prolapse with parturition that accumulates with increasing number of deliveries, and (ii) overt prolapse only with elastase-induced injury.

For many years, it was believed that POP was unique to bipedal species. Although puerperal inversion of the uterus (i.e., organ “inside-out”) is fairly common in sheep and cattle, loss of vaginal, bladder, and rectal support (POP) is uncommon and usually associated with pregnancy and delivery. Whereas POP occurs in nonhuman primates [41], it is uncommon and almost always associated with complicated vaginal delivery. Thus, previous reports of POP in mice with various degrees of elastinopathy represented an unexpected opportunity to investigate the role of dysfunctional elastic fiber homeostasis in the pathogenesis of POP in humans. Previously, we found that (1) Fbln5 is required for assembly and organization of tropoelastin into mature elastic fibers, (2) Fbln5 aids in correct localization of lysyl oxidase-like-1 onto elastic fibers [42], (3) local injection of purified elastase results in postpartum prolapse of the vaginal wall, and (4) the RGD domain of Fbln5 is crucial to inhibit vaginal MMP-9 activity [14]. The results reported herein suggest that the normal elastic fiber network established during development and resultant suppression of MMP activation early in life is sufficient to maintain pelvic organ support in mice even with decreased Fbln5 in the vaginal wall and renders the host quite resistant to pregnancy- and injury-induced prolapse. Impaired Fbln5 function and increases in MMP9 in the vaginal wall may represent merely markers of increased risk for POP.

*Fbln5* knock-in mice in which the RGD domain of Fbln5 was mutated to RGE (*Fbln5*^*RGE/RGE*^) do not develop prolapse spontaneously and exhibit normal elastic fibers in connective tissues, including the vagina [14]. Interestingly, these mice also showed increased levels of MMP-9 in the vaginal wall and modest susceptibility to prolapse with inhibition of lysyl oxidase [14]. Our results in *Fbln5* cKO animals are congruent with findings in *Fbln5*^*RGE/RGE*^ mice emphasizing the crucial role of elastic fiber development in maintenance of pelvic organ support as adults. Using our strategy of generating conditional cell-specific loss of Fbln-5 resulted in only partial loss of this matrix protein in the vagina. Cell types other than vaginal stromal cells contribute to synthesis of Fbln5 and may also play a role in maintenance of vaginal support. For example, Fbln5 secreted from endothelial cells may lead to substantial content of the protein even when absent in α actin-expressing cells. Since the protein is secreted into the matrix, the relative contribution of each component in the vagina is not known. It is possible, that greater depletion of vaginal Fbln5 may lead to a more severe phenotype. Both MMP9 and Fbln5 expression are modulated by TGFβ signaling [43–45], and TGFβ signaling is further activated by MMP9. Although it is well-known that TGFβ contributes to tissue fibrosis through increased expression of matrix components, recent studies indicate that matrix stiffness itself contributes to fibrosis and TGFβ activation [46]. Fbln5 also increases tissue stiffness [46, 47], but also increases the fibrotic phenotype in skin [46]. Thus, we suggest that profound loss of fibulin-5 after elastase injury in CKO mice may result in reduced stiffness of pelvic connective tissue and prolapse of the pelvic organs. Future studies are needed to examine the relationship between fibulin-5 and TGFβ signaling in the vaginal wall.

Acquired decreased Fbln5 in the vaginal wall did not lead to prolapse under baseline conditions even for one year in mice. It is difficult to demonstrate significant impairment of elastic fibers in aging mice, however, simply because of their limited life span. Aside from findings in one report in which Fbln5 was proteolyzed in connective tissues as a function of age [26], most investigators resort to experimentally-induced degradation of elastic fibers to mimic elastic fiber defects associated with aging in humans [48–53]. Our model in which elastase was injected into the vaginal wall tissue revealed that conditional loss of Fbln5 predisposed to pelvic organ prolapse. We suggest that this model may thereby mimic elastic fiber degradation in aging women. Our previous finding in which certain protease inhibitors were decreased [18] whereas elastic fiber proteases were increased [14] in postmenopausal women with prolapse support this concept.

To translate these findings to humans, therefore, anatomical and physiological differences between mice and humans have to be taken into consideration. First, in mice, the pubic ligament undergoes significant remodeling and extension to allow for passage of the fetus. In women, this is not possible because a stable pelvic structure is necessary for bipedal function. Second, the fetal head of mice is small relative to that of human neonates. Finally, labor and delivery is rapid in mice. In women, prolonged distension of the pelvic floor with childbirth is believed to result in hypoxia, generation of free radicals and reactive oxygen species, which can activate matrix metalloproteases and tip the balance to the destruction of elastic fibers. Thus, childbirth in women is inherently more traumatic on connective tissues of the pelvic floor. It follows, therefore, that although severe perturbation of vaginal elastic fibers was necessary to elicit prolapse in cKO mice, women may be more sensitive to reduction of Fbln5 due to gravitational forces on the pelvic floor, more traumatic parturition, and loss of elastic fibers with age.

## References

[1] Wu JM, Matthews CA, Conover MM, Pate V, Jonsson Funk M. Lifetime risk of stress urinary incontinence or pelvic organ prolapse surgery. Obstet Gynecol. 2014;123(6):1201–6. 10.1097/AOG.0000000000000286 24807341 PMC4174312

[2] DeLancey JOL. The hidden epidemic of pelvic floor dysfunction: Achievable goals for improved prevention and treatment. Am J Obstet Gynecol. 2005;192(5):1488–95. 15902147 10.1016/j.ajog.2005.02.028

[3] Olsen AL, Smith VJ, Bergstrom JO, Colling JC, Clark AL. Epidemiology of surgically managed pelvic organ prolapse and urinary incontinence. Obstet Gynecol. 1997;89(4):501–6. 9083302 10.1016/S0029-7844(97)00058-6

[4] Drewes PG, Yanagisawa H, Starcher B, Hornstra IK, Csiszar K, Marinis SI, et al Pelvic organ prolapse in Fibulin-5 knockout mice: pregnancy changes in elastic fiber homeostasis in mouse vagina. Am J Pathol. 2007;170:578–89. 17255326 10.2353/ajpath.2007.060662 PMC1851882

[5] Liu X, Zhao Y, Gao J, Pawlyk B, Starcher B, Spencer JA, et al Elastic fiber homeostasis requires lysyl oxidase-like 1 protein. Nat Genet. 2004;36(2):178–82. . 14745449 10.1038/ng1297

[6] Lee UJ, Gustilo-Ashby AM, Daneshgari F, Kuang M, Vurbic D, Lin DL, et al Lower urogenital tract anatomical and functional phenotype in lysyl oxidase like-1 knockout mice resembles female pelvic floor dysfunction in humans. Am J Physiol Renal Physiol. 2008;295(2):F545–55. 10.1152/ajprenal.00063.2008 18495804

[7] Rahn DD, Acevedo JF, Word RA. Effect of Vaginal Distention on Elastic Fiber Synthesis and Matrix Degradation in the Vaginal Wall: Potential Role in the Pathogenesis of Pelvic Organ Prolapse. American journal of physiology Regulatory, integrative and comparative physiology. 2008;295:R1351–8. 10.1152/ajpregu.90447.2008 18635445 PMC3774207

[8] Wieslander CK, Marinis SI, Drewes PG, Keller PW, Acevedo JF, Word RA. Regulation of elastolytic proteases in the mouse vagina during pregnancy, parturition, and puerperium. Biol Reprod. 2008;78(3):521–8. . 18003950 10.1095/biolreprod.107.063024

[9] Liu X, Zhao Y, Pawlyk B, Damaser M, Li T. Failure of Elastic Fiber Homeostasis Leads to Pelvic Floor Disorders. Am J Pathol. 2006;168(2):519–28. 16436666 10.2353/ajpath.2006.050399 PMC1606509

[10] Rahn DD, Acevedo JF, Roshanravan S, Keller PW, Davis EC, Marmorstein LY, et al Failure of pelvic organ support in mice deficient in fibulin-3. Am J Pathol. 2009;174(1):206–15. 10.2353/ajpath.2009.080212 19095964 PMC2631333

[11] Wieslander CK, Rahn DD, McIntire DD, Acevedo JF, Drewes PG, Yanagisawa H, et al Quantification of pelvic organ prolapse in mice: vaginal protease activity precedes increased MOPQ scores in fibulin 5 knockout mice. Biol Reprod. 2009;80(3):407–14. 10.1095/biolreprod.108.072900 18987327 PMC2805390

[12] Zhang Xuan C LK N WB. Regulation of MMP-9 expression and activity in the mouse uterus by estrogen. Molecular Reproduction and Development. 2007;74(3):321–31. 16967517 10.1002/mrd.20582

[13] Moalli PA, Shand SH, Zyczynski HM, Gordy SC, Meyn LA. Remodeling of vaginal connective tissue in patients with prolapse. Obstet Gynecol. 2005;106(5 Pt 1):953–63. . 16260512 10.1097/01.AOG.0000182584.15087.dd

[14] Budatha M, Roshanravan S, Zheng Q, Weislander C, Chapman SL, Davis EC, et al Extracellular matrix proteases contribute to progression of pelvic organ prolapse in mice and humans. J Clin Invest. 2011;121(5):2048–59. 10.1172/JCI45636 21519142 PMC3083772

[15] Dviri M, Leron E, Dreiher J, Mazor M, Shaco-Levy R. Increased matrix metalloproteinases-1,-9 in the uterosacral ligaments and vaginal tissue from women with pelvic organ prolapse. Eur J Obstet Gynecol Reprod Biol. 2011;156(1):113–7. 10.1016/j.ejogrb.2010.12.043 21277671

[16] Wu JM, Visco AG, Grass EA, Craig DM, Fulton RG, Haynes C, et al Matrix metalloproteinase-9 genetic polymorphisms and the risk for advanced pelvic organ prolapse. Obstet Gynecol. 2012;120(3):587–93. 10.1097/AOG.0b013e318262234b 22914468 PMC3427536

[17] Bump RC, Norton PA. Epidemiology and natural history of pelvic floor dysfunction. Obstetrics & Gynecology Clinics of North America. 1998;25(4):723–46. 9921553 10.1016/s0889-8545(05)70039-5

[18] Budatha M, Silva S, Montoya TI, Suzuki A, Shah-Simpson S, Wieslander CK, et al Dysregulation of protease and protease inhibitors in a mouse model of human pelvic organ prolapse. PLoS One. 2013;8(2):e56376 10.1371/journal.pone.0056376 23437119 PMC3577807

[19] Sharrow L, Tinker D, Davidson JM, Rucker RB. Accumulation and regulation of elastin in the rat uterus. Proceedings of the Society for Experimental Biology & Medicine. 1989;192(2):121–6. 2813441 10.3181/00379727-192-42965

[20] Starcher B, Percival S. Elastin turnover in the rat uterus. Connective Tissue Research. 1985;13(3):207–15. 3159538 10.3109/03008208509152400

[21] Yanagisawa H, Hammer R, Richardson J, Williams S, Clouthier D, Y M.—Role of Endothelin-1/Endothelin-A receptor-mediated signaling pathway in the aortic arch patterning in mice.[see comment].—Journal of Clinical Investigation. 1998;102(1):22–33. 9649553 10.1172/JCI2698 PMC509061

[22] Yanagisawa H, Davis EC, Starcher BC, Ouchi T, Yanagisawa M, Richardson JA, et al Fibulin-5 is an elastin-binding protein essential for elastic fibre development in vivo. Nature. 2002;415(6868):168–71. Epub 2002/01/24. 10.1038/415168a . 11805834

[23] Hsu-Wong S, Katchman SD, Ledo I, Wu M, Khillan J, Bashir MM, et al Tissue-specific and developmentally regulated expression of human elastin promoter activity in transgenic mice. Journal of Biological Chemistry. 1994;269(27):18072–5. 8027067

[24] Koo HP, Macarak EJ, Chang SL, Rosenbloom J, Howard PS. Temporal expression of elastic fiber components in bladder development. Connect Tissue Res. 1998;37(1–2):1–11. . 9643643 10.3109/03008209809028896

[25] Fata JE, Ho AT, Leco KJ, Moorehead RA, Khokha R. Cellular turnover and extracellular matrix remodeling in female reproductive tissues: functions of metalloproteinases and their inhibitors. Cell Mol Life Sci. 2000;57(1):77–95. . 10949582 10.1007/s000180050500 PMC11146958

[26] Hirai M, Ohbayashi T, Horiguchi M, Okawa K, Hagiwara A, Chien KR, et al Fibulin-5/DANCE has an elastogenic organizer activity that is abrogated by proteolytic cleavage in vivo. J Cell Biol. 2007;176(7):1061–71. 10.1083/jcb.200611026 17371835 PMC2064089

[27] Sharma AK, Lu G, Jester A, Johnston WF, Zhao Y, Hajzus VA, et al Experimental Abdominal Aortic Aneurysm Formation Is Mediated by IL-17 and Attenuated by Mesenchymal Stem Cell Treatment. Circulation. 2012;126(11 Suppl 1):S38–45. . 22965992 10.1161/CIRCULATIONAHA.111.083451 PMC3448933

[28] Ailawadi G, Moehle CW, Pei H, Walton SP, Yang Z, Kron IL, et al Smooth muscle phenotypic modulation is an early event in aortic aneurysms. J Thorac Cardiovasc Surg. 2009;138(6):1392–9. 10.1016/j.jtcvs.2009.07.075 19931668 PMC2956879

[29] Azuma J, Asagami T, Dalman R, Tsao PS. Creation of murine experimental abdominal aortic aneurysms with elastase. J Vis Exp. 2009;(29). . 19629030 10.3791/1280 PMC3148686

[30] Buckley C, Wyble CW, Borhani M, Ennis TL, Kobayashi DK, Curci JA, et al Accelerated enlargement of experimental abdominal aortic aneurysms in a mouse model of chronic cigarette smoke exposure. Journal of the American College of Surgeons. 2004;199(6):896–903. 15555973 10.1016/j.jamcollsurg.2004.08.010

[31] Daugherty A, Cassis LA. Mouse models of abdominal aortic aneurysms. Arterioscler Thromb Vasc Biol. 2004;24(3):429–34. . 14739119 10.1161/01.ATV.0000118013.72016.ea

[32] Eskandari MK, Vijungco JD, Flores A, Borensztajn J, Shively V, Pearce WH. Enhanced abdominal aortic aneurysm in TIMP-1-deficient mice. J Surg Res. 2005;123(2):289–93. . 15680392 10.1016/j.jss.2004.07.247

[33] Hannawa KK, Cho BS, Sinha I, Roelofs KJ, Myers DD, Wakefield TJ, et al Attenuation of experimental aortic aneurysm formation in P-selectin knockout mice. Ann N Y Acad Sci. 2006;1085:353–9. . 17182955 10.1196/annals.1383.014

[34] Thompson RW, Curci JA, Ennis TL, Mao D, Pagano MB, Pham CT. Pathophysiology of abdominal aortic aneurysms: insights from the elastase-induced model in mice with different genetic backgrounds. Ann N Y Acad Sci. 2006;1085:59–73. . 17182923 10.1196/annals.1383.029

[35] Otto-Verberne CJ, Ten Have-Opbroek AA, Franken C, Hermans J, Dijkman JH. Protective effect of pulmonary surfactant on elastase-induced emphysema in mice. Eur Respir J. 1992;5(10):1223–30. . 1486969

[36] Kuang PP, Goldstein RH, Liu Y, Rishikof DC, Jean JC, Joyce-Brady M. Coordinate expression of fibulin-5/DANCE and elastin during lung injury repair. American Journal of Physiology Lung Cellular & Molecular Physiology. 2003;285(5):1147. 10.1152/ajplung.00098.2003 12909585

[37] Nguyen AD, Itoh S, Jeney V, Yanagisawa H, Fujimoto M, Ushio-Fukai M, et al Fibulin-5 Is a Novel Binding Protein for Extracellular Superoxide Dismutase. Circulation research. 2004;95(11):1067–74. 15528465 10.1161/01.RES.0000149568.85071.FB

[38] Murakami S, Nagaya N, Itoh T, Iwase T, Fujisato T, Nishioka K, et al Adrenomedullin Regenerates Alveoli and Vasculature in Elastase-induced Pulmonary Emphysema in Mice. Am J Respir Crit Care Med. 2005;172(5):581–9. 15947283 10.1164/rccm.200409-1280OC

[39] Houghton AM, Quintero PA, Perkins DL, Kobayashi DK, Kelley DG, Marconcini LA, et al Elastin fragments drive disease progression in a murine model of emphysema. J Clin Invest. 2006;116(3):753–9. . 16470245 10.1172/JCI25617 PMC1361346

[40] Yao H, Arunachalam G, Hwang JW, Chung S, Sundar IK, Kinnula VL, et al Extracellular superoxide dismutase protects against pulmonary emphysema by attenuating oxidative fragmentation of ECM. Proc Natl Acad Sci U S A. 2010;107(35):15571–6. 10.1073/pnas.1007625107 20713693 PMC2932580

[41] Otto LN, Slayden OD, Clark AL, Brenner RM. The rhesus macaque as an animal model for pelvic organ prolapse. Am J Obstet Gynecol. 2002;186(3):416–21. . 11904600 10.1067/mob.2002.121723

[42] Liu X, Zhao Y., Gao J., Pawlyk B., Starcher B., Spencer J.A., Yanagisawa H., Zuo J., and Li T. Elastic fiber homeostasis requires lysyl oxidase-like 1 protein. Nat Genet. 2004;36(2):178–82. 14745449 10.1038/ng1297

[43] Schiemann WP, Blobe GC, Kalume DE, Pandey A, Lodish HF. Context-specific effects of fibulin-5 (DANCE/EVEC) on cell proliferation, motility, and invasion. Fibulin-5 is induced by transforming growth factor-beta and affects protein kinase cascades. J Biol Chem. 2002;277(30):27367–77. 10.1074/jbc.M200148200 . 12021267

[44] Chou YT, Wang H, Chen Y, Danielpour D, Yang YC. Cited2 modulates TGF-beta-mediated upregulation of MMP9. Oncogene. 2006;25(40):5547–60. 10.1038/sj.onc.1209552 . 16619037

[45] Moalli PA, Klingensmith WL, Meyn LA, Zyczynski HM. Regulation of matrix metalloproteinase expression by estrogen in fibroblasts that are derived from the pelvic floor. Am J Obstet Gynecol. 2002;187(1):72–9. . 12114891 10.1067/mob.2002.124845

[46] Nakasaki M, Hwang Y, Xie Y, Kataria S, Gund R, Hajam EY, et al The matrix protein Fibulin-5 is at the interface of tissue stiffness and inflammation in fibrosis. Nat Commun. 2015;6:8574 10.1038/ncomms9574 26469761 PMC4634219

[47] Rahn DD, Ruff MD, Brown S, Tibbals HF, Word RA. Biomechanical properties of the mouse vagina: changes seen in pregnancy and with elastinopathy. Am J Obstet Gynecol. 2008;198(5):590e1–6. 18455541 10.1016/j.ajog.2008.02.022 PMC2760217

[48] Chapman SL, Sicot FX, Davis EC, Huang J, Sasaki T, Chu ML, et al Fibulin-2 and fibulin-5 cooperatively function to form the internal elastic lamina and protect from vascular injury. Arterioscler Thromb Vasc Biol. 2010;30(1):68–74. 10.1161/ATVBAHA.109.196725 19893004 PMC2800831

[49] Matt P, Huso DL, Habashi J, Holm T, Doyle J, Schoenhoff F, et al Murine model of surgically induced acute aortic dissection type A. J Thorac Cardiovasc Surg. 2010;139(4):1041–7. 10.1016/j.jtcvs.2009.08.039 19910001 PMC3454491

[50] Gonzalez JM, Briones AM, Somoza B, Daly CJ, Vila E, Starcher B, et al Postnatal alterations in elastic fiber organization precede resistance artery narrowing in SHR. Am J Physiol Heart Circ Physiol. 2006;291(2):H804–12. 10.1152/ajpheart.01262.2005 . 16565305

[51] Osman M, Keller S, Hosannah Y, Cantor JO, Turino GM, Mandl I. Impairment of elastin resynthesis in the lungs of hamsters with experimental emphysema induced by sequential administration of elastase and trypsin. J Lab Clin Med. 1985;105(2):254–8. . 2857757

[52] Yamaguchi T, Yokokawa M, Suzuki M, Higashide S, Katoh Y, Sugiyama S, et al The effect of immunosuppression on aortic dilatation in a rat aneurysm model. Surg Today. 2000;30(12):1093–9. . 11193741 10.1007/s005950070007

[53] Morimoto K, Hasegawa T, Tanaka A, Wulan B, Yu J, Morimoto N, et al Free-radical scavenger edaravone inhibits both formation and development of abdominal aortic aneurysm in rats. J Vasc Surg. 2012;55(6):1749–58. 10.1016/j.jvs.2011.11.059 . 22341578

